# Adeno-Associated Virus Vector Mediated Delivery of the HBV Genome Induces Chronic Hepatitis B Virus Infection and Liver Fibrosis in Mice

**DOI:** 10.1371/journal.pone.0130052

**Published:** 2015-06-15

**Authors:** Lei Ye, Haisheng Yu, Chengwen Li, Matthew L. Hirsch, Liguo Zhang, R. Jude Samulski, Wuping Li, Zhong Liu

**Affiliations:** 1 Institute of Blood Transfusion, Chinese Academy of Medical Sciences and Peking Union Medical College, Chengdu, China; 2 Key Laboratory of Immunity and Infection, Institute of Biophysics, Chinese Academy of Sciences, Beijing, China; 3 Gene Therapy Center, Department of Pharmacology, University of North Carolina, Chapel Hill, North Carolina, United States of America; 4 MOH Key Laboratory of Systems Biology of Pathogens, Institute of Pathogen Biology, Chinese Academy of Medical Sciences & Peking Union Medical College, Beijing, China; University of Texas HSC at San Antonio, UNITED STATES

## Abstract

Liver cirrhosis and hepatocellular carcinomas are major health problems of chronic hepatitis B virus (HBV) infection. To date, rare model has reproduced liver fibrosis associated with long-term HBV infection which in turn has hindered both the understanding of HBV biology and the development of new treatment options. Here, using adeno-associated virus serotype 8 (AAV8) mediated delivery of a 1.2-kb HBV genome, we successfully generated a chronic HBV infectious mouse model that presents the associated liver fibrosis observed following human infection. After AAV8/HBV1.2 vector administration, mice demonstrated effective HBV replication and transcription which resulted in HBV antigen expression and viremia over 6 months. Although no obvious acute inflammatory response was noted, these mice still developed chronic liver disease and hepatic fibrogenesis as demonstrated by increased ground glass-like hepatocytes, an increasing trend of collagen deposition and upregulated fibrosis markers, including type I collagen, type III collagen, tissue inhibitor of metalloproteinase (TIMP), and transforming growth factor-β1(TGF-β1). Taken together, AAV-mediated HBV gene delivery to the mouse liver, induced HBV persistent infection accompanied by liver fibrosis which can serve as a model for investigating the precise mechanisms underlying liver fibrosis following chronic HBV infection as well as for the potential development of novel therapeutics.

## Introduction

Approximately 240 million people worldwide are chronically infected with hepatitis B virus (HBV) and a large proportion of chronic infections develop into hepatocellular carcinoma or cirrhosis [1]. These complications often result in liver failure and over one million deaths are reported annually [2–4]. Thus, HBV-related diseases remain a major public health challenge. Chronically-infected patients can be treated with several drugs, including IFN-α and nucleoside analogs such as lamivudine or adefovir. IFN-α regulates the immune response by increasing viral clearance, whereas nucleoside analogs interfere with viral DNA replication. However, the effectiveness of these drugs is limited. And challenges remain in terms of their clinical application, including low efficacy, undesirable side effects, and resistant HBV mutations[5–9]. Thus, there is a need to develop both novel therapeutic reagents that inhibit HBV replication and representative HBV animal models to evaluate new therapeutic strategies.

The key characteristic of hepadnaviruses is their high degree of species-specificity and hepatic tropism, such as lack specific receptors and cellular factors that are needed in viral entry and trafficking which could make mice resistant to HBV infection [10–13]. Up to date, chimpanzees are the only permissive animals that are fully infected to HBV,and acute infections and hepatitis would develop upon inoculation with HBV positive serum; however, these animals do not envolve chronic liver disease, but develop cellular immune response similar to that demonstrated in human being during acute infection [14, 15]. However, several limitations including large size, ethical issues, and high costs restrict their use for basic research and therapeutic drug screening. The tree shrew (*Tupaia belangeri*) is a squirrel-like HBV permissive small animal, but HBV infection only result in mild and transient infections [16]. HBV transgenic mice are used to clarify the HBV replication mechanism and specific oncogenes function [17–20]. However, due to HBV genes integrated into the host genome and expressed as self-proteins and thus, the mice are tolerant to these immunologically antigens and do not induce liver disease. Mouse models using recombinant viral vectors or by hydrodynamic injection of naked HBV genome DNA into hepatocytes were used to investigate immune tolerance to HBV antigens and to study the mechanisms of viral clearance [21–24]. However, acute but not chronic, HBV infection is often induced. Two recent studies used AAV to deliver HBV genome DNA to mouse liver and induced HBV persistent infection and immune tolerance to HBV [25, 26]. Both papers tried to elucidate immune mechanism of the persistence HBV infection. Another study elucidated the mechanisms of chronic HBV infection and immunopathogenesis in a humanized mouse model [27]. Thus, there is a need to develop a novel mouse model of persistent HBV infection to investigate viral pathogenesis and to screen new antiviral drugs.

The present study developed a mouse model of persistent HBV infection by using an AAV vector to transfer the hepatitis B virus genome (HBV1.2) into mouse liver cells which initiated hepatitis B virus production, thereby leading to persistent viremia and the induction of liver fibrosis. This mouse model could be used to elucidate the biological process underlying HBV pathogenesis and to screen small molecules that may be effective for the treatment of chronic HBV infection and liver fibrosis.

## Materials and Methods

### Cell lines

HEK 293T cells and Huh7.5.1 cells were maintained in Dulbecco's modified Eagle's medium (Sigma, St Louis, MO) supplemented with 10% fetal bovine serum and penicillin-streptomycin (100U/ml). The cells were maintained at 37°C in a 5% CO_2_ atmosphere.

### Generation of the AAV-HBV vector (SSV9-1.2HBV)

A pHBV1.2 plasmid containing 1.2 copies of the HBV genome (genotype D) was used to generate the HBV fragment. This fragment was cloned into the pSSV9 vector (which contained the inverted terminal repeat of AAV type 2 at both ends because the *rep* and *cap* genes were exchanged) to generate pSSV9-1.2HBV. Briefly, p-SSV9 was digested with *Xba*l to generate a linear vector and pHBV1.2 was digested with *SacI* and *Hind*III to generate the inserted fragment. The linearized p-SSV9 backbone and the HBV fragment were blunted using Klenow I and then ligated using T4 ligase. Pseudotyped AAV8 vectors were produced in 293T cells using a triple-plasmid transfection protocol and purified with CsCl gradients[28]. The vector titers were determined by quantitative PCR (qPCR).

### In vitro study

Huh7.5.1 cells were seeded into a six well plate at 4×10^5^ per well and cultured at 37°C in a 5% CO_2_ atmosphere. pSSV9-1.2HBV DNA (3μg) was transfected into the Huh7.5.1 cells of each well using Lipofectamine 2000 (Invitrogen, Carlsbad, CA, USA) according to the manufacturer's instructions. PBS was used as control. The supernatant and transfected cells were harvested 72 hrs post-transfection and subjected to nucleic acid extraction using a viral DNA isolation kit (QIANGEN) and then digested with *Dpn*I. The recovered HBV viral DNA was measured via qPCR. In a parallel assay, the supernatant was collected everyday for seven days and replaced with the same amount of media to determine the cumulative abundance of HBsAg and HBeAg by ELISA. At the same time, pSSV9-1.2HBV (10μg) was transfected to Huh7.5 cell (2×10^6^ cell) in 100mm dish. Southern-blot analysis of total DNA extrated from transfected Huh7.5.1 cell and DNA samples extrated from HepG2.2.15 cell and Huh7.5.1 cell as positive control and negtive control respectively. All DNA samples were digested with or without selected restriction enzymes(*EcoR*I or *Hind*III) and were treated with Rnase A before 1.2% gel electrophoresis. The filters were hybridized with a DIG-labeled 3.2kb HBV specific probe.

### Animal study

Normal C57BL/6 mice (aged 6–8 weeks; Sichuan University, Sichuan, China) were bred and maintained at the Laboratory Animal Facility of the Institute of Blood Transfusion, Chinese Academy of Medical Science, Chengdu. Animal care and procedures were performed in accordance with the Guide for the Care and Use of Laboratory Animals, which was approved by the Institutional Animal Care and Use Committee at the Chinese Academy of Medical Science (Permit Number: ILAS-PG-2014-001). All efforts were made to minimize suffering. Mice were injected with the AAV vector [2×10^11^ vector genome equivalents (vg)] in 200μl of phosphate-buffered saline (PBS) via the tail vein. The serum for the ELISAs was prepared from tail bleeding, which was collected in heparinized capillary tubes using standard methods. After dilution with PBS, the serum HBsAg and HBeAg concentrations were measured using an Auszyme Monoclonal Diagnostic ELISA kit (Abbott Laboratories, Abbott Park, IL). Serum alanine aminotransferase (ALT) and aspartate aminotransferase (AST) levels were analyzed using commercially available colorimetric assays (Teco Diagnostics, Anaheim, CA). To collect the livers for the immunohistochemical and nucleic acid analyses, mice were anesthetized using 2.5% avertin and perfused transcardially with cold PBS (pH 7.4), followed by 4% paraformaldehyde in phosphate buffer (0.1 mol/L pH 7.4). Intrahepatic HBcAg and HBsAg were visualized by immunohistochemical staining of OCT-embedded tissues using rabbit anti-HBc and anti-HBs antibodies (Dako, Carpinteria, CA), respectively, and the Envision HRP (diaminobenzidine) system (Dako, Carpinteria, CA). Ten random fields were selected per slide and the percentages of HBsAg- and HBcAg-positive hepatocytes were quantified using Image-Pro Plus software (Media Cybernetics, Rockville, MD). The liver sections were also examined by light microscopy after standard hematoxylin and eosin (H&E) and Masson’s trichrome staining [29, 30]. Sirus red staining of liver sections were observed by polarizingmicroscope.

### qPCR and reverse transcription (RT)-qPCR

To measure the DNA levels of HBV and the AAV vector, nucleic acids were extracted using DNeasy Blood and Tissue Kits (Invitrogen, Carlsbad, CA) according to the manufacturer’s instructions and stored at -80°C prior to PCR analyses. A qPCR standard curve was generated using 10-fold dilutions of the SSV9-1.2HBV plasmid (1.0×10^3^–1.0×10^9^copies/ml). To measure the mRNA levels of HBV, *Timp-1*, and *Tgf-β1*, total RNA was isolated using a NucleoSpinRNA II kit (Macherey Nagal,GmbH & Co. KG, Germany) and reverse transcribed using a First Strand cDNA Synthesis Kit (Toyobo, Japan). All of the qPCR reactions were performed in triplicate in 96-well optical reaction plates using an ABI 7900 Sequence Detection System (Applied Biosystems, Foster City, CA) and SYBR Green I PCR mix (Roche Diagnostics, Indianapolis, IN) as previously described [31]. The primer sequences are shown in the S1 Table.

### ELISA

The levels of collagen I and III in the liver and serum samples were determined using commercially available ELISA kits (Mouse Collagentype I (Col I) ELISA kit and Mouse Collagentype III (Col III) ELISA kit); (R&D Systems, Minneapolis, MN). The serum levels of TIMP-1 and TGF-β1 were also determined using commercially available ELISA kits (R&D Systems, Minneapolis, MN). To prepare liver samples, up to100 mg of tissue was homogenized in 200μl of PBS and centrifuged at 2000–3000g for 20 min. The supernatant was collected and analyzed using an ELISA kit.

### Southern and Northern blotting

HBV replicative DNA intermediates and viral RNA were detected by Southern and Northern blot analysis of total genomic liver DNA and RNA respectively with DIG High Prime DNA Labeling and Detection Starter Kit II and DIG Northern Starter Kit (Roche Diagnostics, Indianapolis, IN). 3.2kb HBV genome was labeled by DIG as southern blot probe. For northern blotting, PCR products were used for labeling and synthesized DIG labeled RNA as northern blotting probes. GAPDH (house keeping gene) sequence from homo sapiens and rattus were amplified by RT-PCR and used to normalize the amount of RNA bound to the membrane.

### Statistical analysis

Data were expressed as the mean ±SD. Statistical analysis was performed using two-way analysis of variance (ANOVA, Graphpad prism 5) to determine statistically significant differences between groups. P<0.05 was considered statistically significant.

## Results

### In vitro characterization of the recombinant AAV-HBV vector

**Fig 1:**
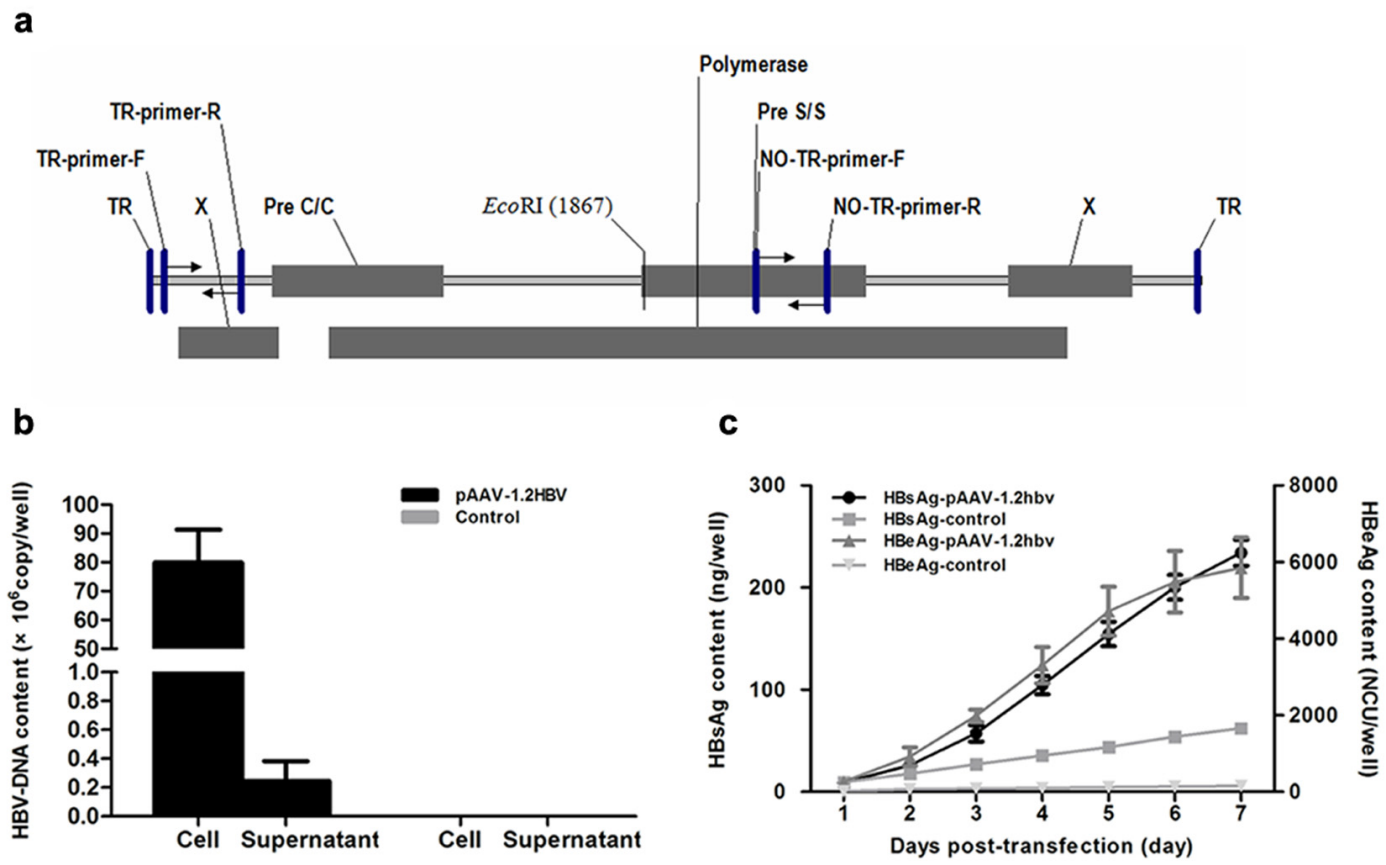
Schematic illustration of the AAV vector used to transfer the HBV genome and in vitro determination of the replication ability of the construct. (a) A fragment containing 1.2 copies of the HBV genome (genotype D) was generated from the pHBV1.2 plasmid and cloned into the p-SSV9 vector, which contained the ITR of AAV type 2 at both ends by exchanging the rep and cap genes (see Materials and Methods). The q-PCR primers were indicated. (b, c) Plasmid of pSSV9-1.2HBV (3μg) was transfected to Huh7.5.1 cell and determined the HBV viral genomes contents and/or the cumulative expression of HBsA and HBeAg in the cell and/or supernatant respectively, by Q-PCR and ELISA. Data represent the mean ±SD (n = 4).

**Fig 2:**
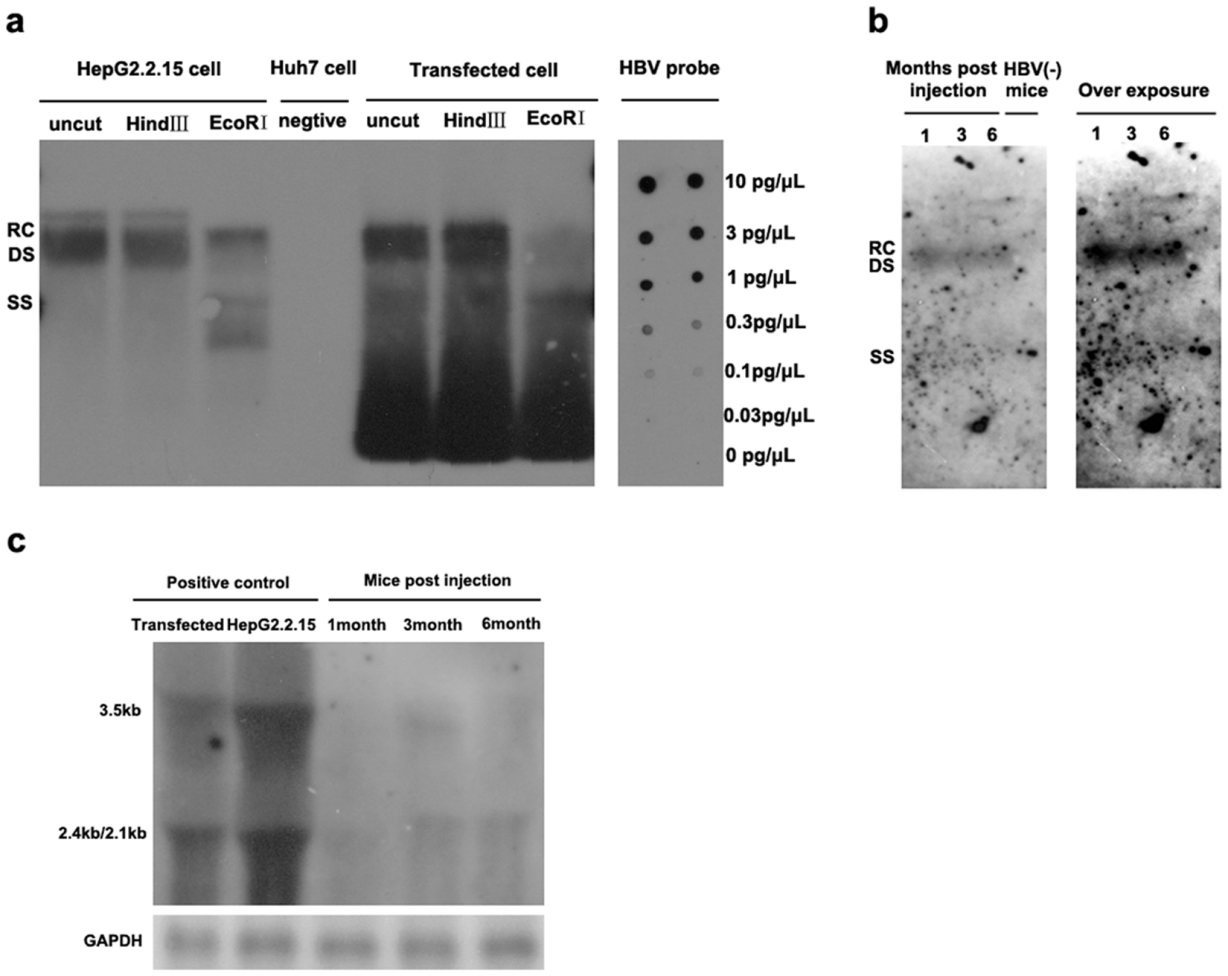
Southern and Northern blot analysis of HBV replication and transcription in vitro and in vivo. (a) Detection of HBV DNA replicative intermediates 7 days post pSSV9-1.2HBV transfected to Huh7.5.1 cell. (b) Intermediates of HBV DNA replication 1, 3, 6 months after AAV8-1.2HBV injection. The relaxed-circular(RC), double-stranded linear (DS) and single-stranded (SS)DNAs were indicated.(c) Northern blotting to detect 3.5kb pregenomic, 2.4kb and 2.1kb mRNAs in the livers post injection. The amount of RNA bound to the membrane was normalized by housekeeping gene (GAPDH). HBV(-) mice, PBS injected mice.

HBV genomes have been delivered into the livers of mice via hydrodynamic injection and by using an adenoviral vector; however, these methods can lead to the rapid clearance of the HBV genomes. Alternatively, HBV-transgenic mice have been used to model HBV infections, however these mice are tolerant to the viral antigens and the persistent expression of self-antigens limits their use for evaluating antiviral drugs. To overcome these limitations, the present study attempted to generate a mouse model that faithfully mimics chronic HBV infection in humans. As mice cannot be directly infected by HBV due to the lack of the HBV receptor required for viral uptake, mice were injected with an AAV vector harboring HBV1.2 genomic DNA, which has been used previously to induce HBV replication in both mouse hepatocytes and human HepG2 cells[32, 33]. AAV8 is highly efficient in transducing mouse liver, so this serotype was used to mediate HBV gene transfer in the present mouse model. As shown in Fig 1a, a fragment that comprised 1.2 copies of HBV (genome D) was cloned into the p-SSV9 vector, which contained the ITR of AAV type 2 at both ends. To verify the HBV production and gene expression of the construct, pSSV9-1.2HBV was transfected into Huh7.5.1 and the amount of viral genome DNA and HBsAg and HBeAg expression was evaluated. As indicated in Fig 1b, HBV viral genome DNA was produced both in supernatant and in cells three days after transfection (Fig 1b). However, the intracellular viral DNA content was approximately 300 times higher than that found in the supernatant (Fig 1b). As shown in Fig 1c, HBsAg and HBeAg were produced at increasing trend overtime and secreted into the supernatant of the transfected cell. At the same time, southern blot analysis of total DNA extracted from transfected Huh7.5.1 cell showed relaxed-circle (rc) DNA, ds DNA and ssDNA (Fig 2a). *EcoR*I restriction enzyme (restriction enzyme cutting site included in HBV genome) and *Hind*III restriction enzyme (restriction enzyme cutting site was not included in HBV genome) were chosen to digest samples in order to cut high molecular weight DNA strands into smaller fragments. Southern blot analysis of uncut total DNA sample was similar to *Hind*III treated sample, but different from *EcoR*I treated sample. HBV relaxed-circle (rc) DNA and ds DNA were digested in *EcoR*I treated sample. These data verified that pITR2-1.2HBV induces HBV replication and gene expression in Huh7.5.1 cell.

### AAV vector mediates HBV genome transfer, replication, and transcription in mouse liver

**Fig 3:**
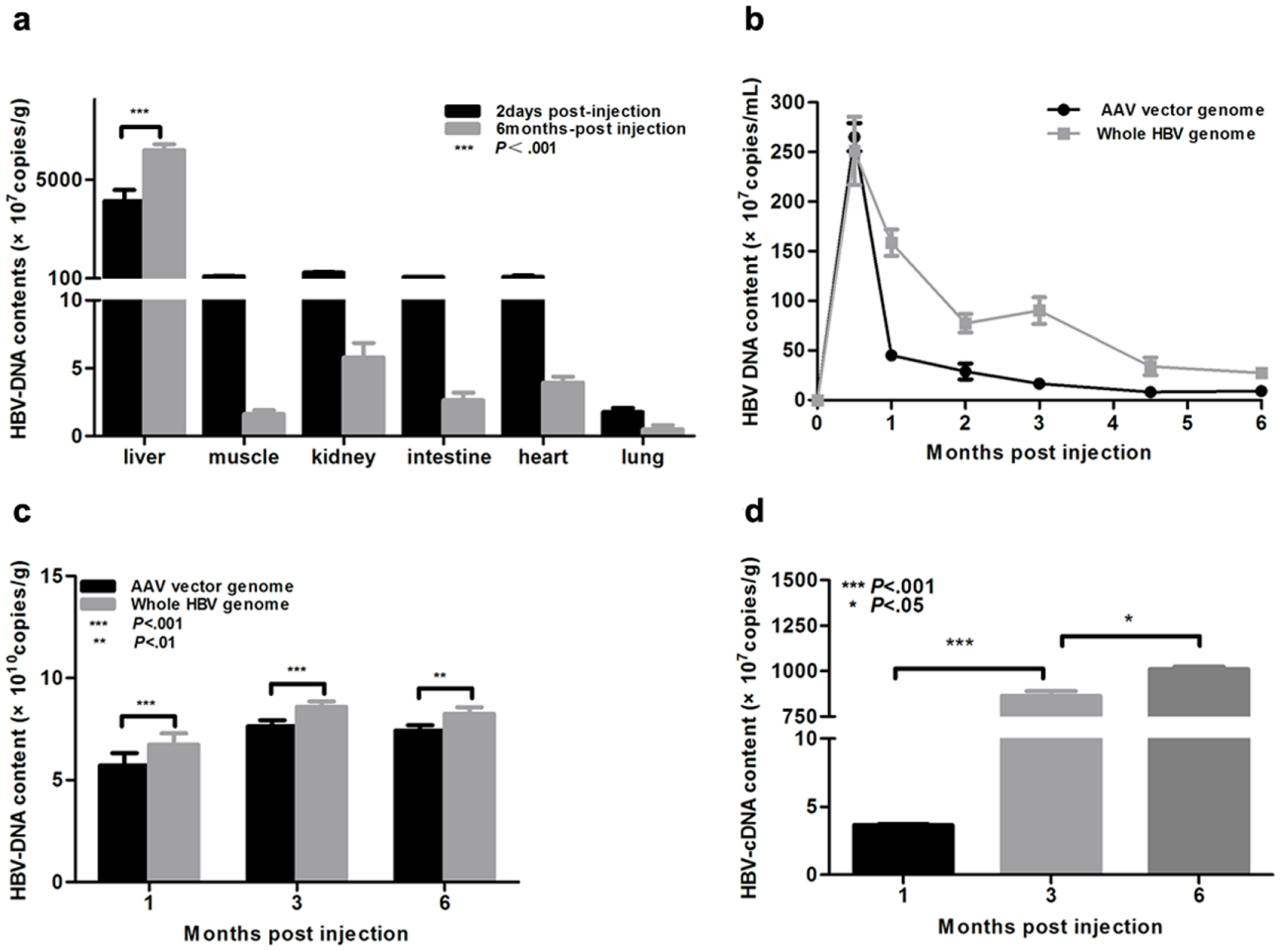
AAV-HBV-mediated efficient HBV gene transfer, replication, and transcription in mouse liver. Mice were injected intravenously with the AAV-HBV vector (2 × 10^11^viral genome equivalents (vg)) and then bled or sacrificed at the indicated time points. (a) HBV viral genomes in selected tissues at 2 days and 6 months after injection. (b, c) Levels of AAV vector and whole HBV genome in serum (b) and liver (c) samples. HBV viremia is expressed as the difference between the whole HBV genome content and the AAV vector genome content. (d) Reverse transcription quantitative PCR analysis of the HBV cDNA content of the liver. Statistical analyses were performed using a two-way analysis of variance. Data represent the mean ±SD (n = 4).

Following the in vitro plasmid vector verification, AAV8 vectors that harbor the 1.2HBV genome were produced in 293T cells as described previously[28]. Then, mice were injected intravenously with the AAV8-1.2HBV vector (2×10^11^vg). Two days post-injection (p.i.), over 90% of the HBV DNA was distributed in the liver (Fig 3a). At 6 months p.i., the HBV DNA levels in the liver had increased by 40% compared to two days p.i., whereas the levels were reduced markedly in all other tissues examined (muscle, kidney, intestine, heart, and lung). This suggests that the HBV genome replicated in mouse liver. Two pairs of qPCR primers (S1 Table) were used to quantify the HBV genome copy number. The first pair, which included a forward primer located in the AAV2 ITR and a reverse primer located in the HBV X region, was used to quantify the AAV vector DNA. The second pair of forward and reverse primers targeted in the HBV S region and was used to quantify the HBV genome (including the AAV vector, replicated HBV, and HBV packaged virus). First, the HBV genome copy number was examined in the serum at 0–6 months p.i.. The viral genome levels peaked at 2 weeks p.i., before decreasing rapidly as they were cleared from the serum (Fig 3b). This was followed by a slow decrease in genome abundance after 2 months. At the 2 week time point, the HBV genome and AAV vector levels detected by the two sets of primers were approximately equal; however, there was a 2–5-fold difference at the later time points (Fig 3b), suggesting that the HBV virus was secreted into the serum. The HBV genome content of the mouse liver was also measured using the same primer pairs. At the 1, 3, and 6 month time points, the overall HBV genome content was significantly higher than that of the AAV vector (Fig 3c). There was also an increasing trend at the first three months, which suggested that the HBV genome replicated in the mouse liver. During 1–6 month post AAV8-1.2HBV injected, HBV replication intermediates(rc DNA, ds DNA and ss DNA) were also observed in C57BL/6 mice by Southern blot analysis (Fig 2b), but covalently closed circular DNA was not detected. These results demonstrated that encapsulated HBV viral DNA was produced and that physiologically relevant viremia was present in the mouse model. Transcription of HBV in the mouse liver was also examined by RT-qPCR and Northern blot. Compared to the 1 month time point (3.65 ×10^7^ copies/g), the amount of viral cDNA was 200-fold higher at 3 months p.i., reaching 1.01×10^10^ copies/g at 6 months p.i. (Fig 3d). And northern blot analysis of total Hepatic RNA extracted from injected mice, 3.5kb, 2.4kb and 2.1kb HBV transcripts were detected correlated with the expression of HBsAg in serum (Fig 2c). This confirmed that transgene transcription occurred following AAV-1.2HBV vector delivery.

### In vivo HBV gene expression levels and kinetics

**Fig 4:**
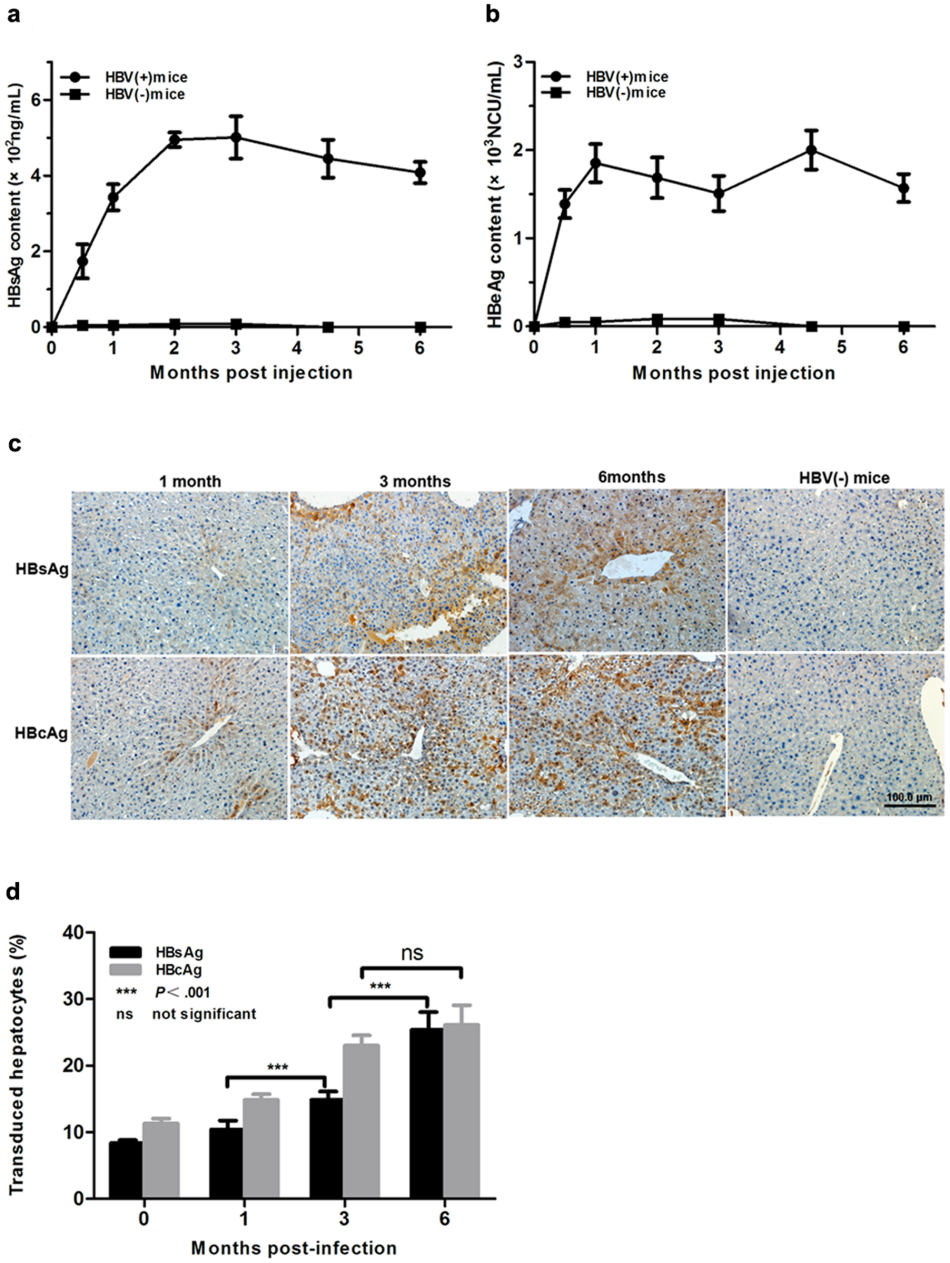
AAV-HBV-mediated efficient gene transduction *in vivo*. (a) Serum HBsAg was measured in a quantitative ELISA and the average ng/ml serum was plotted at the indicated times p.i.. Data represent the mean ±SEM. (b) Serum HBeAg was measured in a quantitative ELISA and the average NCU/ml serum was plotted at the indicated times p.i.. Data represent the mean ±SEM. (c) Immunohistochemical staining of HBsAg and HBcAg in liver sections. Staining was performed in tissue sections from four animals per group. Representative sections are shown. (d) Quantification of the immunohistochemical data shown in (c).Ten random fields were selected per slide and the percentages of HBsAg- and HBcAg-positive hepatocytes were quantified using Image-Pro Plus (Media Cybernetics, Rockville, MD). Statistical analyses were performed using two-way analysis of variance. HBV(-) mice, PBS injected mice.

Viral replication and viremia were detected in AAV-1.2HBV infected mice, but it was not sufficient for an adequate experimental model of HBV infection. Thus, AAV-mediated gene transduction in the serum and liver were analyzed by ELISA and immunohistochemical staining for the HBV antigen, respectively. The results showed that AAV8-1.2HBV injection led to a persistent infection, which was characterized by the presence of HBsAg and HBeAg, or HBcAg in the mouse serum and/or liver for 6 months. The secretion of viral antigens into the blood was monitored over time. HBV surface antigen was detectable at 2 weeks p.i., peaking at 4.85×10^2^ng/ml at 2 months. After this, the serum HBsAg level declined slowly and reached a plateau concentration of 4.0×10^2^ng/ml at 6 months p.i. (Fig 4a). The expression profile of HBeAg showed a similar trend, except that it peaked at 1 month p.i. (Fig 4b). Interestingly, the serum level of HBsAg remained relatively steady over the course of 6 months and there was a lack of seroconversion to anti-HBs (both IgM and IgG). Immunohistochemical staining was used to detect the expression of HBsAg and HBcAg in the livers of mice treated with AAV8-HBV1.2. As shown in Fig 4c, similar to the serum expression profile, HBsAg-positive hepatocytes were distributed randomly throughout the liver at 1 month p.i. and most visual fields contained no stained cells, although some rare hepatocytes showed light staining of the cytoplasm. However, the expression of HBsAg showed an increasing trend during the 6 months period, which may have reflected a cumulative effect. The expression of HBcAg occurred rapidly, which was obvious throughout the liver at 1 month p.i., and increased significantly at 3 months p.i. before remaining relatively stable. By contrast, cells from the HBV(-) group exhibited no staining. These results demonstrate that the AAV vector mediated HBV transgene production.

### AAV8-1.2HBV does not generate obvious acute inflammation, but induces liver fibrosis accompanied by chronic liver injury

**Fig 5:**
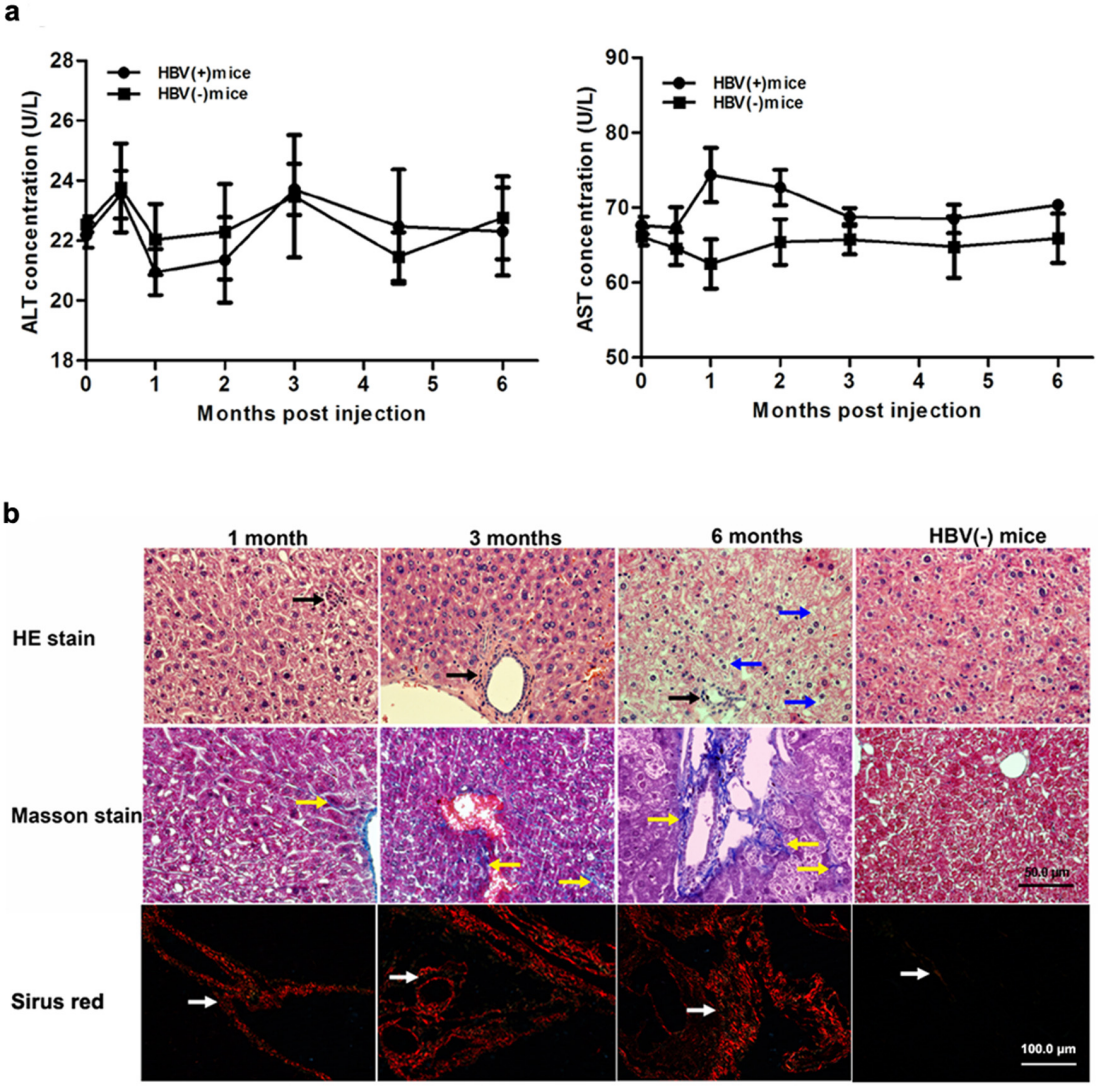
Injection AAV-HBV induces chronic liver injury but not acute inflammation. Serum and liver samples were collected from HBV(+) and HBV(-) mice at the indicated time points. (a) ALT and AST levels. (b) Liver samples stained with hematoxylin and eosin to detect inflammatory responses and with Masson’s stain and Sirus red stain to detect collagen deposition. Black arrows, inflammatory cell infiltration; blue arrows, fat vacuoles; yellow and white arrows, collagen deposition. HBV(-) mice, PBS injected mice.

ALT and AST are enzymes located in liver cells that are released into the circulation by necrotic hepatocytes. Therefore, we monitored these transaminase concentrations in the serum to assess the toxicity following AAV8-1.2HBV vector administration. Compared with HBV(-) mice, AAV8-1.2HBV infection did not increase the serum ALT levels over the course of 6 months (Fig 5a), whereas the serum AST level increased modestly at 1 and 2 months p.i.; however, this difference was not statistically significant. Animals treated with AAV8-1.2HBV did not exhibit other symptoms of systemic toxicity (data not shown). The increased AST levels at 1 and 2 months p.i. may have been associated with HBV transgene expression. These results demonstrate that there was no obvious acute inflammatory response after AAV8-1.2HBV injection.

**Fig 6:**
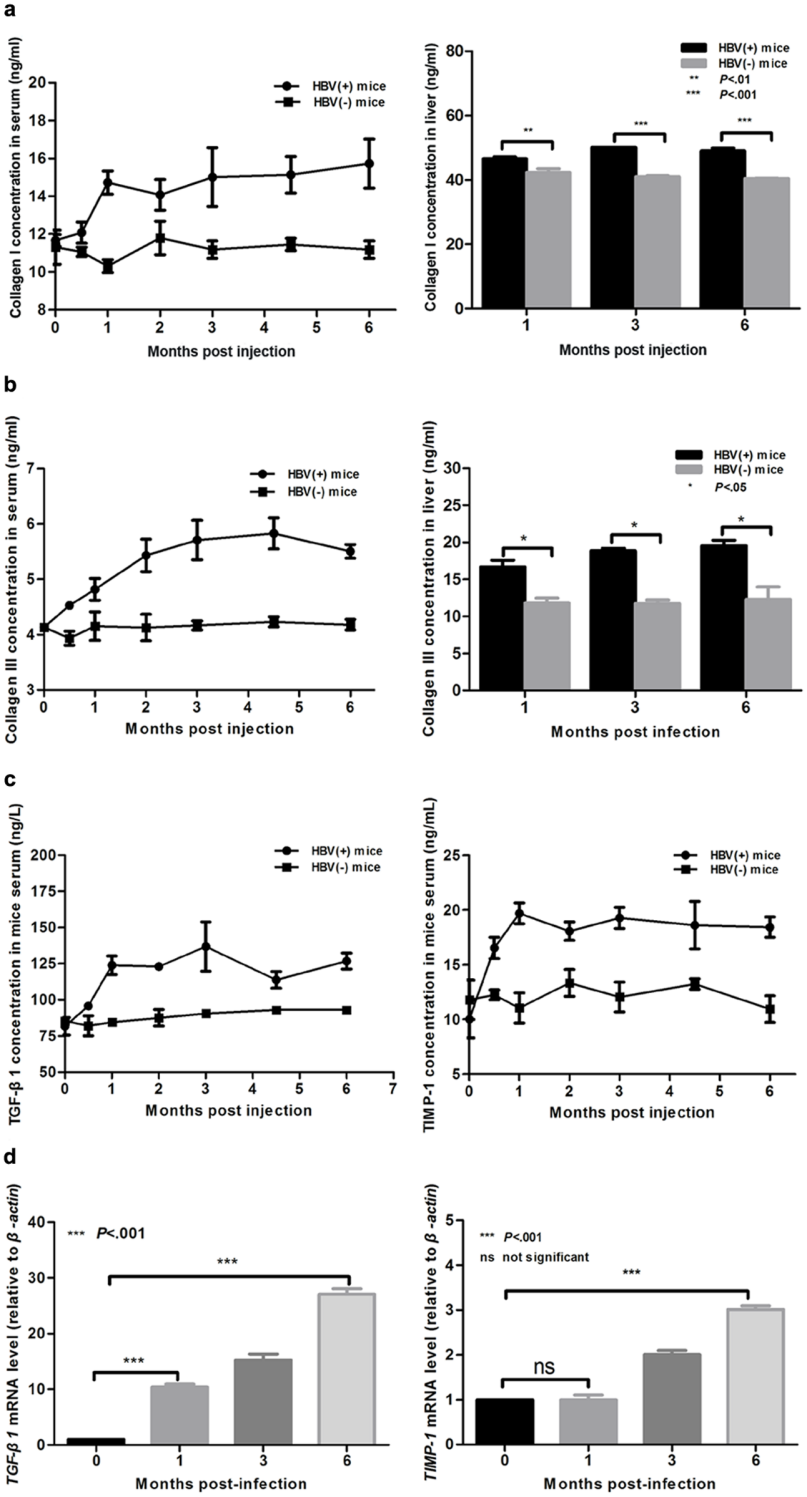
Fibrosis-related markers were significantly upregulated in AAV-HBV injected mice. (a, b) ELISA measurements of the collagen I (a) and III (b) levels in serum and liver samples. (c) ELISA measurements of the TGF-β1 and TIMP-1 protein levels in the serum. (d) Reverse transcription quantitative PCR measurements of *Tgf-β1* and *Timp-1* mRNA levels in the liver. Statistical analyses were performed using a two-way analysis of variance. Data represent the mean±SD (n = 4). HBV(-) mice, PBS injected mice.

Previous studies show that the chronic inflammation related to HBV infection contributed to liver fibrosis in human patients[34, 35]. To investigate whether liver fibrosis and chronic liver injury were present following AAV81.2HBV transduction, histopathological changes in liver sections were analyzed overtime by H&E, Masson’s staining and Sirus red stain. As shown in Fig 5b, mild inflammation and hepatic necrosis were indicated. A mild inflammatory cell infiltration surrounding the portal area (black arrow) was shown by H&E staining at 1, 3, and 6 months p.i., and most of hepatocytes were normal up to 3 months p.i.. At 6 months p.i., however, the hepatic lobular structure was marked damage and ground glass-like hepatocytes were indicated, macrovesicular steatosis degeneration (blue arrow) was also observed and the vascular and portal areas was obviously broadened. Collagen (stained blue by Masson’s staining and red by Sirus red stain; yellow and white arrow) deposition was observed by an increasing trend during the study period (Fig 5b). Proliferated fibers were stained blue in liver by Masson stain, and proliferated collagen I fibers were stained red by Sirus Red stain in liver (Fig 5b). The levels of collagen I and III in the serum and liver of the model mice were determined to facilitate a quantitative assessment of the major extracellular matrix proteins. Compared with normal mice at 1 month p.i., model mice showed a 20–45% increase in collagen I (Fig 6a) and a 30–70% increase in collagen III (Fig 6b) in the serum and the liver, respectively. ELISA and RT-qPCR were next used to examine the expression of fibrosis related proteins and genes, respectively. The levels of TGF-β1 and TIMP-1 protein (Fig 6c) and mRNA (Fig 6d) were significantly higher in HBV(+) mice than in HBV(-) mice. These results suggest that AAV-HBV injection did not induce a serious acute inflammatory response, whereas it did induce fibrosis and chronic liver injury.

## Discussion

The present study investigated the use of an AAV vector to transfer the HBV genome into mouse liver cells. Mice were injected with the AAV8-1.2HBV vector via the tail vein, which initiated HBV production with persistent antigenemia, viremia and hepatic fibrosis with no obvious acute inflammation. HBV replication, transcription, and expression persisted for more than 6 months. The viremia level was similar to that previously reported for an adenovirus vector-mediated mouse model [20, 21] and a transfection mouse model established via hydrodynamic injection of naked DNA [22]. The small animal model is desirable for evaluating the effects of antiviral treatment and has several advantages over other mouse models. For example, in the HBV-transgenic mouse model, the HBV genome cannot be eliminated due to integrate into the host genome. In other work, Adenoviral vectors carrying a 1.3-fold HBV genome to mouse liver, and HBV replication was established successfully, and HBV viremia was detectable in the serum; however, the persistent infection using AdHBV vectors is severely restricted by the immune response against the vectors capsid [21]. In the hydrodynamic mouse model, only a small ratio of mouse hepatocytes were transduced and the viral replication rate persisted low. The HBV replication levels also decreased after 7 days in immunocompetent mice, and HBV was already eliminated from the blood 1 week later [22].

In the present study, AAV8-1.2HBV infection successfully constructed a model of persistent HBV infection (prolonged high-level viremia and antigenemia). There was little or no acute infection or liver inflammation, as indicated by normal transaminase levels and low levels of lymphocyte infiltration (Fig 5b); however, the mice did develop chronic liver disease, demonstrated by the presence of ground glass-like hepatocytes like that was observed in the chronically-infected patients livers[36–38]. This is very like the pathogenesis in adults; patients develop persistent infection with HBV after acute infection and evolve chronic infection with different degrees of severity[39, 40]. Generally, the liver diseases is usually thought caused by the immune response against viral antigens but not virus itself [40, 41]. There was no direct cytopathic effects on hepatocytes by HBV infection by the presence of a lot of asymptomatic chronic HBV carriers with no signs of liver injury[42]. Previous studies found defects of the immune response in HBV chronic infected patients, including reduction of HBV-specific antibodies[43, 44], delayed lymphocytes migration, and increment of suppressor lymphocytes[45]. Circulating HBsAg is probably the mechanism of defective immune response in HBV carriers[43, 46]. Further studies are needed to identify the immunological factors that responsible for the development of pathogenesis during persistent HBV infection in this mouse model and to identify the correlation between liver fibrosis and the immune response.

Liver fibrosis is a utmost pathway to most chronic liver diseases and evolvements as a consequence of liver damage caused by viral, parasitic, toxic, metabolic, and or autoimmune disorders[47]. The stylemark of liver fibrosis is the increment in the degree and composition of the extracellular matrix, which leads to the deposition of collagen I. Hepatic stellate cells post an important role in liver fibrogenesis[48–50]. Several factors could stimulate resting hepatic stellate cells to an active fibrogenic cell type. Activated hepatic stellate cells generate fibrotic components, including collagen type I, inhibitors of matrix degradation such as TIMP-1, and other growth factors (TGF-β1, etc.)[51]. The novel mouse model described herein showed up-regulation of fibrotic markers, such as type I collagen and type III collagen, in the liver and serum. TIMP-1 is a critical factor regarded to fibrogenesis and can prohibit the degradation of the extracellular matrix, and its expression leads to the deposition of extracellular matrix in models of liver fibrosis[52]. TIMP-1 was also significantly increased in the serum of the model mice in the present study. TGF-β1 plays a major role in the homeostasis of fibrogenesis processes, and its expression was up-regulated and maintained throughout the fibrotic process in the present study.

In summary, we have developed a model of persistent HBV infection that induced liver fibrosis in immunocompetent mice, which resembles that in patients with chronic HBV infection. Although previous studies have established mouse models of liver fibrosis[53–55], no current animal model mimics liver fibrosis during long-term HBV infection in immune competent mice. Therefore, this new model may be useful for investigating the precise mechanisms that underlie liver fibrosis during chronic HBV infection, and may facilitate the development of new therapeutics.

## Supporting Information

S1 Table Primer sequence used in this study. (DOCX)
