# Supplementary material for: Adeno-Associated Virus Vector Mediated Delivery of the HBV Genome Induces Chronic Hepatitis B Virus Infection and Liver Fibrosis in Mice
Source: PLoS One. 2015 Jun 15;10(6):e0130052. doi: 10.1371/journal.pone.0130052 (PMC4468063; doi:10.1371/journal.pone.0130052)
Supplement: S1 Table — (DOCX) [file pone.0130052.s001.docx]

**S1 Table. Primer sequence used in this study**

| **Primer name** | **Target gene** | **Sequence (5′→3′)** |
| --- | --- | --- |
| TR-primer-F | AAV ITR | AACCCGCCATGCTACTTATCTACGT |
| TR-primer-R | HBV X | CACACAGTCTTTGAAGTAGGCC |
| NO-TR-primer-F | HBV S | GCCTCATCTTCTTGTTGGTTC |
| NO-TR-primer-R | HBV S | GAAAGCCCTACGAACCACTGAAC |
| RT-q-βactin-F | β-actin | CGTTGACATCCGTAAAGACC |
| RT-q-βactin-R | β-actin | TAGAGCCACCAATCCACACA |
| RT-q-TGF-β1-F | TGF-β1 | CTGCTGACCCCCACTGATAC |
| RT-q-TGF-β1-R | TGF-β1 | GTGAGCACTGAAGCGAAAGC |
| RT-q-TIMP-1-F | TIMP-1 | ATTTGCACATCACTGCCTGC |
| RT-q-TIMP-1-R | TIMP-1 | GGGATGGCTGAACAGGGAAA |
